# Abstracts From the Argentinian Society of Osteology and Mineral Metabolism (AAOMM) and Argentinian Society of Osteoporosis (SAO) 2020 Annual Meeting

**DOI:** 10.1002/jbm4.10553

**Published:** 2021-09-29

The Argentinian Society of Osteology and Mineral Metabolism (AAOMM) and the Argentinian Society of Osteoporosis (SAO) are the two national scientific societies dedicated to clinical and basic research into mineralized tissue. Meetings of each society are held annually, attracting a wide audience from throughout Argentina, Latin America, and beyond. During 2020, AAOMM and SAO held the 3rd Joint Congress of Osteology, which traditionally is balanced between clinical, therapeutic, and basic science. The participation of young scientists and clinicians is actively encouraged. This year, a leader team in musculoskeletal clinical and basic science organized the Annual Meeting. The composition of the Scientific Committee was Dr. Pablo Costanzo (SAO), Dr. María Diehl (AAOMM), Dr. Evangelina Giacoia (SAO), Dr. Diana Gonzalez (AAOMM), Dr. María Silvia Larroudé (SAO), and Dr. Gabriela Picotto (AAOMM). The composition of the Organizing Committee was Dr. María Lorena Brance (AAOMM), Dr. Graciela Brito (AAOMM), Dr. Vanina Farias (SAO), Dr. María Laura García (SAO), Dr. María Susana Moggia (SAO), Dra. Paula Rey (AAOMM), and both current presidents Dr. Lucas R. Brun (AAOMM) and Dr. José Luis Mansur (SAO). The program brought together international leaders such as Dra. Geraldine Altamar (Colombia), Dra. Teresita Bellido (USA), and Dr. Adolfo Diez Pérez (Spain).

## Postsurgical permanent hypoparathyroidism: can we anticipate it? A cohort study

### Garcia ML, Lisdero AP, Otero MJ, Segarra AE, González Pernas M, Mumbach GA, Genovesi E, Speroni R

#### Endocrinology Department, Sanatorio Dr Julio Méndez, Buenos Aires, Argentina

Hypoparathyroidism (HypoPTH) is a disease manifested by hypocalcemia, hyperphosphatemia and inappropriate or low level of parathyroid hormone (PTH). Common causes are thyroid or parathyroid surgery. HypoPTH could be classified: permanent (P) or transient (T). P‐HypoPTH is the feared complication associated with high morbidity in follow‐up. Recognizing which patient has a higher risk of developing this unwanted complication will help us to achieve an earlier and prompt treatment. Objective: Predict P‐HypoPTH in postsurgical (PS) patients obtaining a cutoff value before 6 months follow‐up. Methods: PS patients from Endocrinology Registry of Sanatorio Dr. Julio Mendez (Argentina) were retrospectively followed for 12 months. We defined P‐HypoPTH as hypocalcemia needing calcium supplements and vitamin D for more than 6 months postsurgery. Calcium (Ca), PTH, phosphorus, vitamin D, magnesium, and requirement of supplementation were measured a day after, a week, and every 3 months. Two groups were formed and compared by Mann Whitney *U* test, logistic regression, receiver operating characteristic (ROC) curves were performed. Data is expressed by variable distribution, OR with 95% confidence interval (CI); *p* < 0.05 is considered significant. Results: Seventy‐two patients were included. After several ROC curves we determined a predictive diagnostic triad for P‐HypoPTH: week Ca level of 8.9 mg/dL under ≥3 g of calcium supplements +third month phosphorus level > 4 mg/dL; reporting standard error (SE) of 86%–78%, respectively, AUC: 0.87. Conclusion: P‐HypoPTH is an unwanted PS complication. Identifying predictive data to determine which patient is at a higher risk to develop it will help us improve and quicken treatment response. We found multiple risk variables and propose a diagnostic triad constructed by Ca level of 8.9 mg/dL under ≥3 g of calcium supplements +third month phosphorus level > 4 mg/dL, but further studies with a larger number of patients is needed to confirm or improve our findings adding a better sensitivity and specificity.

## Osteogenic bioactivity from third generation biomaterial for regenerative medicine

### Garcia Moreno MF,^1,2^ Martin A,^3^ Manavella G,^4^ Feldman S,^5^ Missana LR ^1,2^

#### ^*1*^ *PROIMI‐IMMCA‐CONICET, Tucumán, Argentina*

#### ^*2*^ *Exp Path Lab & Tissue Eng. Oral Path. Dent School*

#### ^*3*^ *School of Agronomy and Animal Husbandry, Tucumán University, Tucumán, Argentina*

#### ^*4*^ *Oral and Maxillary Surgery, Dental School, Tucumán University*

#### ^*5*^ *LABOATEM ‐ Medical School, Rosario University, Argentina*

The bioengineered matrices were manufactured in our laboratory, combining natural biopolymer and recombinant human parathyroid hormone (rh‐PTH) 1‐34. Third generation biomaterial to stimulate a specific cellular response and promoting in situ bone regeneration were made. The aim was to evaluate in vivo osteogenic ability, applied into critical bone calvariae defects (CBD). Twenty rabbits, anesthetized, received 15‐mm‐diameter (Ø) CBD. They were divided into control group (CG) and experimental group (EG); the last group receive the treatment. They were euthanized at 45 and 90 days, respectively. Cone beam computed tomography (CBCT) study was performed in both groups. Histopathological studies, with decalcification and staining by hematoxylin and eosin (H&E) and Masson's trichrome were made. Digital images obtained by Sony SC‐50 camera adapted to Olympus microscope, with Soft CellSens 1.16, were morphometrically evaluated by Image ProPlus software. Statistical analyzes were performed. At 90 days, histological and morphometrical results shows in CG at CBD area, fibrous scar tissue with scattered bone formation (22%). However, EG shows bone formed at CBD almost entirely (85%). Also, new bone formed adopted appearance like normal (ie, waving, folds, sutures, spaces). The newly bone formed was like compound bone (Masson stain). Significant differences were found between groups (*p* < 0.05) by Mann‐Whitney test. High and significant correlation coefficients were obtained by Pearson and Spearman correlation: ρP = 0.9406 (*p* ≤ 0.001), ρS = 0.8570 (*p* ≤ 0.001). High bone regenerative capacity was demonstrated about this bioengineered matrix. CICUAL‐UNT. 23/2017. Grants PDTS N12, PIP864, PIUNTJ615.

## Dismobility syndrome in community‐dwelling older women

### Abdala R, Crespo Amaya G, Zanchetta MB

#### Instituto de Diagnóstico e Investigaciones Metabólicas (IDIM), Argentina

Introduction: Dismobility syndrome (DS) was recently described. It is characterized by three or more of the following criteria: osteoporosis, falls in the preceding year, low muscular mass, slow gait speed, low grip strength, and obesity or high fat mass. Objectives: To assess the prevalence of DS in a population of women ≥60 years in the city of Buenos Aires, and to determine if there is a relationship with osteoporotic fractures and falls. Materials and Methods: Cross‐sectional study; observational and analytical design. A total of 250 women were recruited consecutively. Body composition and bone mineral density was controlled by DXA (Lunar Prodigy Advance). Falls and comorbidities were assessed with a questionnaire. Muscle strength was measured with hand dynamometry (JAMAR). Measures of physical performance (sit‐stand and walking speed) were recorded using a chronometer. The diagnosis of DS was based on the criteria proposed by Binkley et al. We defined baseline DS as having three or more of the following six factors: appendicular lean mass/height < 5.45 (kg/m^2^); high fat was defined as percentage values above 40% of fat mass; lumbar spine or hip (femur neck or total proximal femur) *T*‐score value ≤ −2.5; gait speed with values below 1.0 m/s; and lower muscle strength (handgrip <20 kg). Exclusion criteria: Women with diabetes, neurologic, or oncologic conditions. Statistics: The samples with normal distribution were evaluated by Student's *t* and Wilcoxon tests for independent samples. Qualitative variables were analyzed using chi‐square. Statistical significance is considered with a *p* < 0.05. Results: The average age of the population was 70.4 ± 7.7 years. Seventy‐seven (29%) met the DS criteria; 57% met three criteria and 34% met four. Women with DS were older (72 versus 69 years), had lower bone mineral density (BMD) at the femoral neck (0.728 versus 0.766 g/cm^2^) and total hip (0.769 versus 0.801 g/cm^2^), lower muscle mass in arms and legs (muscle mass index [IMME] 5.6 versus 5.95 kg/m^2^). The strength was 18.8 versus 23.8 kg and the physical performance tests were lower. All these differences were statistically significant. There was no difference in serum values of 25‐hydroxyvitamin D (32.2 versus 30.4 ng/mL). Women with DS had a higher prevalence of osteoporotic fractures (42% versus 11%) and falls in the last year (60% versus 19%); *p* < 0.0001. Conclusion: Women with DS reported a higher frequency of falls in the last year and a higher prevalence of osteoporotic fractures. The evaluation of these criteria is a useful tool to prevent adverse health events.

## Effects and mechanism of action of quercetin in normal and tumor osteoblasts

### Lezcano V, Principe G, González Pardo V, Morelli S

#### INBIOSUR‐CONICET, Universidad Nacional del Sur. Bahía Blanca, Bs As. Argentina

Estrogens play a key role in regulation of bone mass. Currently, global trends attempt to use natural bioactive compounds such as phytoestrogens to supplement anti‐resorptive drugs effects by modulating cells involved in bone formation. In this work, we studied the effects of the phytoestrogen quercetin (QUE) in normal and tumor osteoblastic cells. Normal osteoblasts cells (hFOB cell line) treated with QUE (1–100μM, 48 hours) indicated that 1μM of QUE significantly increased cell number (143.17 ± 4.27% versus control) and viability (115 ± 3.4% versus control) by trypan blue and MTS assays, respectively. In addition, wound healing and cellular adhesion assays also revealed that QUE (1μM) significantly stimulated both parameters on osteoblasts. Osteoblast differentiation was evaluated in bone marrow stem cells cultured in osteogenic medium by measuring alkaline phosphatase activity, calcium deposition and collagen levels through colorimetric methods. Dose‐response studies showed an increase in these differentiation markers after 0.01μM of QUE. Western blot analysis indicated that QUE (0.01μM, 24 hours) increased phospho‐AKT(Ser473), promoted its target protein GSK3β inactivation and increased β‐catenin expression with nuclear translocation. Instead, dose‐response tests on osteoblastic osteosarcoma cells (ROS 17/2.8) showed that QUE (20–100μM) decreased cell number and viability. Maximum concentration of QUE (100μM) provoked a decrease in phospho‐AKT(Ser473) and phospho‐BAD (pro‐apoptotic protein) and an increase in phospho‐ERK1/2. ERK activation may lead to osteosarcoma cell death because pretreatment with PD98059 inhibitor reverted the QUE effect. Altogether, these results indicate that QUE has anabolic effects on normal osteoblasts and anti‐tumor effects on osteosarcoma cells, being an interesting alternative for bone treatments.

## Angiogenic potential of ionic dissolution products from a new bioactive glass–ceramic material

### Haro Durand L,^1^ Gimeno M,^2^ Gomez Gramajo F,^3^ Vargas G,^3^ Vera Mesones R,^3^ Fanovich A,^4^ Boccaccini A,^5^ Perone M,^6^ Gorustovich A^7^

#### ^*1*^ *IByME*

#### ^*2*^ *IBIOBA*

#### ^*3*^ *Unas*

#### ^*4*^ *INTEMA*

#### ^*5*^ *FAU*

#### ^*6*^ *IIMT*

#### ^*7*^ *UCASAL, Argentina*

This study aimed to evaluate the in vivo neovascularization of Geltrex implants enriched with ionic dissolution products (IDPs) from scaffolds obtained from 45S5 bioglass containing 2% B_2_O_3_ (45S5.2B) under experimental diabetes mellitus (DM). IDPs were obtained by incubating scaffolds for 72 hours in Dulbecco's phosphate‐buffered saline (DPBS). Type I DM was established using the adoptive transfer model in NOD‐scid mice, assigned to the following groups: control group (*n* = 5), non‐compensated DM group (*n* = 5), and insulin‐compensated DM group (*n* = 5). Under anesthesia, the mice were dorsolaterally sc injected with 500 μL of Geltrex containing DPBS enriched with IDPs in combination with basic fibroblast growth factor (bFGF). DPBS + bFGF was used as positive control. In each animal, the positive control was injected on one side and the problem sample on the other side. At 7 days posttreatment, the mice were euthanized and the Geltrex masses removed and then processed for further histological evaluation and flow cytometry. The microscopic study demonstrated the neovascularization of the implants enriched with the IDPs from the 45S5.2B scaffolds in the control and compensated DM mice. Flow cytometry results showed an 85% increase in the number of CD31 + CD45− endothelial cells in Geltrex samples enriched with the IDPs from the 45S5.2B scaffolds implanted in the insulin‐compensated DM group compared to the response observed in the non‐compensated DM mice, which showed a marked decrease (72%) in the number of endothelial cells with respect to the control animals. The results obtained may have therapeutic relevance in DM patients with critical limb ischemia, because the IDPs from 45S5.2B scaffolds could act as inorganic agents with the ability to enhance tissue neovascularization.

## Biocompatibility and bone tissue regeneration using recombinant scaffolds

### Aimone M,^1^ Rodríguez Fonseca A,^2^ Camal Ruggieri IN,^1^ García LA,^1^ Venegas Rojas FM,^1^ Chullo Llerena V,^1^ Ramallo M,^1^ Santiago O,^1^ Lembo IM,^1^ Landgraf T,^1^ Benevidez de Oliveira G,^1^ Farez N,^1^ Gatti D,^4^ Stur M,^4^ Missana LR,^3^ Alonso M,^2^ Rodriguez Cabello JC,^2^ Feldman S^1^

#### ^*1*^ *LABOATEM, Rosario National University, Argentina*

#### ^*2*^ *BIOFORGE, Valladolid University ‐ CIBER‐BBN, Spain*

#### ^*3*^ *Proimi‐Biotecnología, IMMCA‐CONICET, Argentina*

#### ^*4*^ *Diagnóstico por imágenes FCM UNR, Argentina*

Elastin‐like recombinamers (ELR) are biomaterials derived from Val‐Pro‐Gly‐X‐Gly pentapeptide found in elastin, obtained using recombinant DNA technology. Our aim was to investigate if a hydrogel based on ELRs designed by us (HG), regenerated bone tissue, being biocompatible. Two ELRs were designed: RGD cell adhesion sequences (HRGD6), and metalloproteinases (HE5) sensitive sequences, and were cloned into vector pET‐25b (+) for expression in *Escherichia coli* (BLR‐DE3). Side chains of the component lysines were modified to contain azide groups in HRGD6 and cyclo‐octino groups in HE5. Each ELR was dissolved in PBS and mixed with each other forming a covalent crosslinking HG. Cell viability (CV) assays were performed by culturing encapsulated HG fibroblasts, with LIVE/DEAD kit. New Zealand 4‐month‐old rabbits (*n* = 12) were randomly assigned to control (Co) or injury (I), 10 mm diameter in each parietal calvarial bone, implanting 200 μL of HG in left injury (li); right injury (ri), without implant. Clinical controls (Cli) as well as biochemical studies, (Bio: hemogran and transaminases), were performed daily during the first week and 15, 30, and 90 days postsurgery, (CH). Tomographic studies were done on day 90 (To). Postmortem histopathological studies (HS) were done on decalcified samples. CV was 99%. No differences in Cli nor Bio were detected comparing Co versus I. To li showed presence of mineralized tissue compatible with bone tissue with no tissue retraction whereas ri showed very small diffuse areas with increased density. HS of li evidenced the formation of multiple islets of reticular bone that joined each other, a neoformation process similar to intramembranous ossification was distinguished, whereas ri showed fibroblastic‐fibrous tissue. HG showed high cell viability, biocompatibility, and the ability to promote regeneration of missing tissue in an in vivo model. These integrally analyzed results support the potential use of HG for bone tissue regenerative medicine applications.

## SLPI and vitamin D: indirect markers of the immune system in the elderly?

### Oliveri B,^1^ Ambrossi N,^2^ Bonanno M,^1^ Mastaglia S,^1^ Chuluyan E^2^

#### ^*1*^ *Laboratorio Osteoporosis y Enfermedades Metabólicas Óseas, Hospital de Clínicas, Instituto de Inmunología, Genética y Metabolismo (INIGEM‐CONICET‐UBA)*

#### ^*2*^ *CEFYBO‐CONICET, Facultad de Medicina, Universidad de Buenos Aires, Buenos Aires, Argentina*

Vitamin D (VD) has immunomodulatory properties. Immunosenescence and VD deficiency coexist in the elderly, and this could affect immune response mediators such as secretory leukocyte protease inhibitor (SLPI), a leukocyte inhibitor with antimicrobial and anti‐inflammatory activity and promoter of tolerogenic response. Objective: To evaluate circulating SLPI levels as a marker of tolerogenic immune profile, and their association with serum 25‐hydroxyvitamin D (25OHD) levels in the elderly. Materials and Methods: The study comprised 36 women aged (mean ± SD) 70.7 ± 6.9 years. Exclusion criteria: Infectious diseases (within 3 months prior to enrollment), autoimmune diseases, creatinine clearance (CrCl) <60 mL/min, cancer, immunosuppressive drug therapy. Plasma levels of SLPI (ELISA), 25OHD (RIA Diasorin), and CrCl (calculated according to the Crockoft‐Gault formula) were determined. The group with 25OHD <10 ng/mL (*n* = 18) was compared to groups showing adequate levels >30 ng/mL (*n* = 18) (Mann‐Whitney and Wilcoxon). Results: As compared to the group with adequate VD levels (25OHD: 37.4 ± 5.6 ng/mL) (*p* = 0.0001), the group with severe VD deficiency (25OHD: 8.8 ± 1.3 ng/mL) showed lower SLPI levels (9.9 ± 5.3 versus 17.8 ± 6.0 ng/mL, respectively) (*p* = 0.0001), and a positive correlation was observed between SLPI and 25OHD levels (*r* = 0.524, *p* = 0.001). Conclusions: The obtained results confirm that VD deficiency has a negative impact on immune response, promoting a pro‐inflammatory rather than a tolerogenic state.

## Positive association of yerba mate (*Ilex paraguariensis*) consumption with bone mineral density in postmenopausal women by DXA and 3D modeling

### Brun LR,^1,2^ Henríquez M,^3^ Brance ML,^2^ Conforti AS,^4^ Cusumano M,^2^ Gallo ME,^4,5^ Meneses NL,^2^ Saraví FD ^3,5^

#### ^*1*^ *Laboratorio de Biología Ósea, FCM, UNR*

#### ^*2*^ *Reumatología y Enfermedades Óseas, Rosario*

#### ^*3*^ *FUESMEN, Mendoza*

#### ^*4*^ *OSEP, Mendoza*

#### ^*5*^ *FCM, UNCuyo, Mendoza, Argentina*

Mate tea drinking has been associated with higher hip bone mineral density (BMD) in postmenopausal women. We aimed at corroborating this finding and to analyze the relative contribution of cortical and trabecular bone to the higher BMD. We included 300 postmenopausal women: 147 who did not drink mate tea (control) and 153 who had consumed at least 1 L/d during the last 5 years (mate). Women with conditions known to affect hip BMD (other than age and menopause) were excluded. BMD was measured in the left hip with dual‐energy X‐ray absorptiometry (DXA) (GE Lunar Prodigy). Three‐dimensional (3D) modeling was performed with 3D‐Shaper software v. 2.9 (Galgo Medical, Spain). There was no significant difference between groups in either age (*p* = 0.735) or body mass index (BMI) (*p* = 0.082). The mate group had higher total hip BMD (0.942 ± 0.137 g/cm^2^ versus 0.870 ± 0.147 g/cm^2^) and higher 3D variables: integral volumetric BMD (vBMD) (305.5 ± 49.8 mg/cm^3^ versus 279.4 ± 49.6 mg/cm^3^), cortical spine BMD (sBMD) (151.9 ± 22.0 mg/cm^2^ versus 141.3 ± 22.1 mg/cm^2^), and trabecular vBMD (control 151.9 ± 22.0 mg/cm^3^ versus 141.5 ± 37.4 mg/cm^3^); *p* < 0.0001 for all comparisons. Linear regression showed that total hip BMD and 3D variables were positively correlated with BMI in both groups (*p* < 0.001) with similar slopes, but the elevations were consistently higher in the mate group (*p* < 0.001). Present results indicate a positive association of mate tea consumption with BMD for any BMI within the sample range. Cortical and trabecular components of femoral bone mineral make a proportionally similar contribution to the higher total hip BMD in mate tea drinkers.

## Influence of cell phones on bone mineralization: preliminary results

### Triay D,^1^ Saraví FD ^1,2^

#### ^*1*^ *FCM, UNCuyo, Mendoza*

#### ^*2*^ *FUESMEN, Argentina*

We formerly reported differences in hip bone mineral density (BMD) and bone mineral content (BMC) associated with carrying cell phones in males. This study aimed at further study of the possible effects, this time including both males and females. Dual‐energy X‐ray absorptiometry (DXA) scans of both hips were performed in young volunteers of both sexes free from conditions known to affect BMD, with a GE Lunar Prodigy Advance device. Results are mean ± SD unless otherwise stated. Data from 25 subjects (11 males) are available (out of a planned total of 60). Because of the small sample, no analysis according to sex was performed. Age was 27.4 ± 6.2 years, height 163 ± 10 cm, body weight 67.9 ± 15.2 kg, and body mass index (BMI) 25.5 ± 4.2 kg/m^2^. Twenty subjects carried the phone on the right side and five on the left, during a median time of 3.2 years (95% confidence interval [CI] 2.5–5.1 years). Right femoral neck BMD 0.992 ± 0.121 g/cm^2^; left 1.008 ± 0.119 g/cm^2^. Right total hip BMD 1.008 ± 0.137 g/cm^2^; left 1.018 ± 0.138 g/cm^2^. Right trochanter BMD 0.800 ± 0.136 g/cm^2^; left 0.801 ± 0.138 g/cm^2^. Right femoral neck BMC 4.83 ± 0.94 g; left 4.84 ± 0.96 g. Right total hip BMC 31.71 ± 6.99 g; left 31.72 ± 7.02 g. Right trochanter BMC 10.91 ± 5.81 g; left 10.81 ± 5.8 g. Differences in BMD between the hip near the phone versus the opposite hip were 15.92 ± 52.45 mg/cm^2^, 16.36 ± 43.17 mg/cm^2^, and 14.16 ± 36.92 mg/cm^2^ for femoral neck, total hip, and trochanter, respectively. None of the differences was statistically significant. These preliminary results do not support the postulated effect of cell phones previously reported. However, the small sample and different study design might be confounding factors.

## Evaluation of bone health in patients with multiple sclerosis

### Ulla MR,^1^ Vrech C,^2^ Peralta López E,^3^ Castro MJ,^1^ Martos F,^1^ Rivoira MA ^3^

#### ^*1*^ *Fundación ILAIM, Córdoba*

#### ^*2*^ *Sanatorio Allende, Córdoba*

#### ^*3*^ *Facultad de Ciencias Médicas, UNC, Córdoba, Argentina*

Multiple sclerosis (MS) is an autoimmune disease of the central nervous system (CNS). Given the high rate of falls among people with MS, the association between falls and fractures, as well as the use of corticosteroids for acute treatment, our main aim in the work was to evaluate phosphocalcic metabolism and bone mineral density (BMD) in patients with MS and compare them with those of healthy controls. An observational, prospective and open‐label case‐control study was performed that evaluated serum levels of calcium, phosphorus, magnesium, alkaline phosphatase, parathyroid hormone (PTH), osteocalcin, β‐cross laps, and vitamin D. BMD was studied in the femoral neck, total hip, and lumbar spine. Thirty‐six patients with relapsing‐remitting MS (RRMS) (8 men and 28 women) between 20 and 59 years old and 36 healthy patients of similar age and sex were included. All the women were premenopausal. The patients had an expanded disability status scale lower than 5.0 and all had received a corticosteroid pulse in the year prior to bone densitometry. A survey of physical activity, sun exposure, and dairy consumption was also carried out. Both groups presented similar values of calcium, phosphatemia, magnesium, PTH, osteocalcin, and β‐cross laps. In contrast, patients with RRMS had lower serum levels of vitamin D and alkaline phosphatase than controls. BMD was similar in patients with RRMS and in healthy controls in all the body areas analyzed. The *Z*‐score was also similar in both groups. Patients with RRMS performed more physical activity whereas dairy consumption was low but similar in both groups. In relation to sun exposure habits, 70% of MS patients reported inadequate exposure. In our group of patients with MS the levels of vitamin D and alkaline phosphatase were lower than in healthy patients (the levels of vitamin D were lower than recommended). BMD was similar in both groups evaluated; this could be partly due to greater physical activity in patients with MS, which could emphasize the importance of physical activity to maintain BMD. We believe that bone health should be addressed as part of a comprehensive care in MS.

## Pilot study 3D‐DXA: effect of denosumab on the cortical and trabecular bone compartments of the femur

### Bonanno MS,^1^ Gonzalez D,^2^ Bagur A,^2^ Zeni SN,^1^ Oliveri B^1,2^

#### ^*1*^ *Laboratorio de osteoporosis y enfermedades metabólicas óseas, INIGEM‐CONICET‐UBA*

#### ^*2*^ *Mautalen Salud e Investigación, CABA*

Bone densitometry (dual‐energy X‐ray absorptiometry [DXA]) measures bone mineral density (BMD; g/cm^2^) in two dimensions (2D) without differentiating the trabecular and cortical compartments. The 3D‐Shaper software provides a three‐dimensional (3D) reconstruction of the proximal femur with separate analysis of both compartment. Objective: To analyze retrospectively with 3D‐DXA the left femur of osteoporosis (OP) patients treated with denosumab (Dmab). Methodology: A total of 27 women with lumbar spine (LS), femoral neck (FN), and total femur (TF) (GE Lunar Prodigy) BMD before treatment (b) and after receiving two to three doses of 60 mg of Dmab every 6 ± 1 months (s) were analyzed with 3D‐DXA (v. 2.4 Galgo Medicals) measuring: cortical superficial density (CsBMD; mg/cm^2^), volumetric BMD cortical (vBMDC; mg/cm^3^), volumetric BMD trabecular (vBMDT; mg/cm^3^), and volumetric BMD integral (vBMDi; mg/cm^3^). The percentage change (Δ%) was calculated. Statistical analysis: Infostat 2018. Results (mean ± SD; *p < 0.05; **p < 0.001): age 66.6 ± 10.5 years; 96.3%: prior OP treatment (88.9% bisphosphonates); 35.7% had a history of fractures. b: cross‐linked C‐telopeptide (CTX) 295 ± 138; b‐alkaline phosphatase (b‐ALP) 59 ± 13; bone Gla‐protein (BGP) 20 ± 9.2. BMDb (*T*‐score): LS 0.960 ± 0.127 (−1.97 ± 1.05); FN 0.707 ± 0.059 (−2.38 ± 0.43); TF 0.717 ± 0.074 (−2.31 ± 0.59). 3D‐DXA b: CsBMD 122.37 ± 13.42; vBMDC 700.08 ± 66.39; vBMDT 105.96 ± 21.65; vBMDi 237.85 ± 34.50. Δ%s: CTX −73.2**; b‐ALP −21.6**; BGP ‐56.5**. BMDs Δ% (*T*‐score): LS +4.3**(−1.67 ± 1.11**); FN +3.3**(−2.22 ± 0.44**); TF +2.7** (−2.16 ± 0.56**). 3D‐DXAs (Δ%): CsBMD +1.32*; vBMDC +0.53 (NS); vBMDT +5.3**; vBMDi +2.36**. FN and TF BMD Δ% correlated with 3D measurements Δ%: +0.66 to +0.92**. Conclusions: In our cohort an increase in 3D‐DXA parameters were observed with Dmab treatment, predominantly in the trabecular compartment. The LS, FN, and TF BMD significant increment correlated positively with changes of both trabecular and cortical components of 3D‐DXA analysis.

## Cone‐beam computed tomography (CBCT) to evaluate microarchitecture of ewe jawbone after estrogen withdrawal and biphosphonate treatment

### Avendaño ME,^1^ Poletto A,^1^ Davison MR,^2^ Bonanno MS,^3^ Zeni SN ^3^

#### ^*1*^ *Diagnóstico por Imágenes I, Fac de Odontología, UNCUYO*

#### ^*2*^ *Carrera de Odontología, UNRN*

#### ^*3*^ *Lab Osteopatías Metabólicas, INIGEM, Argentina*

Cone‐beam computed tomography (CBCT) is a noninvasive three‐dimensional (3D) reconstruction technique that enables the assessment of jawbone microarchitecture. It is used to illustrate results through 3D images. In the present report, the combination of CBCT with other techniques was used to evaluate changes in composition and microarchitectural structure quantity of the ewe jawbone after estrogen withdrawal (OVX) and/or chronic treatment with high doses of zoledronic acid (ZOL). Three groups of adult Corriedale ewes (35–40 kg) were used: OVX: OVX ewes receiving saline solution (SS); ZOL: OVX ewes treated with ZOL (4 mg/month) for 28 months for high cumulative dose of ZOL in bones. SHAM: SHAM ewes receiving SS. At the end of the study, hemimandibles were extracted, bone mineral density (BMD) and bone mineral content (BMC) of the mandibles were evaluated ex vivo by dual‐energy X‐ray absorptiometry (DXA) (Lunar DPX) and CBCT (Planmeca Promax 3D Classic). Results CBCT: OVX versus SHAM significantly decreased BMC and BMD (*p* < 0.001); bone volume/total volume (BV/TV%), trabecular thickness (Tb.Th), connectivity, and anisotropy (*p* < 0.0075, *p* < 0.0075, *p* < 0.001, and *p* < 0.02, respectively) while trabecular spacing (Tb.Sp) (*p* < 0.0002) was significantly increased. ZOL did not show statistical differences when compared to SHAM. ZOL showed values of anisotropy significantly higher than OVX (*p* < 0.018) and Tb.Sp significantly lower than OVX (*p* < 0.043). BV/TV%, Tb.Th, and connectivity as compared to OVX showed a clear tendency to be higher and almost reached significance (*p* = 0.055, *p* = 0.061, and *p* = 0.054, respectively). Maxillary BMC and BMD were lowest in OVX (*p* < 0.05) and were significantly higher in ZOL than in SHAM (*p* < 0.05). Conclusion: The micro–computed tomography (CT) technique was useful to evaluate the deterioration of the bone quality by estrogen withdrawal and the recovery by ZOL treatment.

## Experimental periodontitis: effects of a low dose of PTH 1–34 for a short time

### Davison MR,^1^ Bidevich NL,^1^ Preliasco M,^1^ Bonanno MS,^2^ Zeni SN ^2^

#### ^*1*^ *Carrera de Odontología, UNRN*

#### ^*2*^ *Laboratorio de Osteopatías Metabólicas, INIGEM, FFyB‐UBA, Hospital de Clínicas (UBA/CONICET)*

Periodontitis (P) is chronic disease that induces a progressive bone resorption. Parathyroid hormone (PTH) administration has anabolic and anti‐inflammatory effects, properties necessary to achieve bone recovery. P can be experimentally induced, via placement of cotton ligatures in the gingival sulcus around the molar teeth that increase biofilm accumulation and disruption of the gingival epithelium, leading to bone loss. We investigated whether intermittent administration of a low dose of PTH 1‐34 during a short period of time in rats having experimental P would block alveolar bone (AB) loss. P was induced in 16 female Wistar rats (221 ± 15 g). Ligature was replaced every 4 days. Rats were randomly divided in two groups and subcutaneously injected every 48 hours for 18 days with: saline solution (G1) and 1.2 μg PTH 1‐34 (G2) in the alveolar sulcus. Eight rats were left as healthy controls. After euthanasia, the hemimandibles were extracted and fixed in formalin buffer for histological analysis of subchondral tibia (ST) and AB bone volume (BV/TV%), and periodontal space height (PSH). Results (mean ± SD of C, G1, and G2 respectively): BV/TV% ST: 38.77 ± 2.59; 38.29 ± 3.9 and 37.75 ± 1.45; BV/TV% AB: 50.3 ± 3.6^c^; 35.6 ± 4.3^a^ and 42.0 ± 1.4^b^; PSH (mm): 0.196 ± 0.057^a^; 0.809 ± 0.115^b^ and 0.706 ± 0.065^c^. Different letters: *p* < 0.05. ST BV/TV evidenced no systemic effect of PTH treatment. The trabecular AB in G2 showed a significant recovery whereas a small recovery in PSH was observed versus C. However, a great percentage of osteoid tissue as compared to G1 rats was observed (data not shown). Conclusion: The decrease in AB loss and the increment in osteoid formation suggested that intermittent low dose of PTH for a short time induced tissue regeneration that attenuated P AB loss. Grant: PI UNRN 40‐A‐467.

## Prenatal exposure to fluoride impairs bone strength and increases osteocyte connexin43 expression in mandibula of suckling rats

### Interlandi V,^1^ Fontanetti P,^1^ Gallará R,^1^ Ponce R,^1^ Rigalli A,^2^ Centeno V^1^

#### ^*1*^ *Departamento de Biología Bucal, Facultad de Odontología, UNCórdoba*

#### ^*2*^ *Laboratorio de Biología Ósea, Facultad de Ciencias Médicas, UNRosario, Argentina*

In previous works we demonstrated that chronic exposure to sodium fluoride (NaF) during early stage of bone formation increases mandibular bone volume and bone mineral density (BMD) and impairs dental eruption. It is known that the connexin 43 (Cx43) expression in osteocytes constitutes one of the main regulators of its activity and controls its viability. The aim of our work was to analyze the effect of maternal exposure to NaF on the mandibular bone biomechanical properties of suckling rats. For this, offspring from two groups of mothers were used: (a) controls and (b) treated (NaF 50 mg/L). The treatment was performed 30 days prior to mating, during pregnancy and lactation. Animals (*n* = 3–6 per group) were euthanized at 15 days old by cervical dislocation. Mandibles were removed and processed to measure biomechanical properties and histomorphometric assays. Trabecular bone strength was evaluated by a compression assay in 2.5‐mm mandibular transversal sections. Number of osteoblasts (N.Ob/mm^2^) and osteocytes (N.Ot/mm^2^) were quantified in buccolingual sections of cancellous bone underlying the developing first mandibular molar stained with hematoxylin and eosin (H&E). Cx43 protein expression was analyzed using immunohistochemistry. Results were expressed as mean ± standard error (SE) and analyzed by Student's *t* test. We observed a decrease in material mechanical parameters in mandibles of 15‐day‐old pups exposed to F^−^. No statistic differences were observed in the structural mechanical parameters of mandibles from 15‐day‐old pups by the F^−^ treatment. The N.Ob/mm^2^ and N.Ot/mm^2^ increased in 15‐day‐old pups exposed to F^−^ (*p* < 0.05). Immunoreactivity of Cx43 was higher in osteocytes from F^−^‐exposed animals than in those from controls (*p* < 0.05). The data suggest that F^−^ exposure during early stage of bone formation alters the mandibular bone quality in 15‐day‐old pups decreasing the resistance to bone strain; this correlates with the increment of the protein Cx43 expression in osteocytes of the mandible.

## Normal and hypercalcemic primary hyperparathyroidism evaluation by DXA and 3D modeling

### Karlsbrum S,^1^ Costanzo P,^1^ Di Gregorio S,^2^ Salerni H^1^

#### ^*1*^ *CICEMO, Buenos Aires, Argentina*

#### ^*2*^ *Cetir/Ascires, Barcelona, España*

Elevated parathyroid hormone (PTH) and consistently normal serum calcium may represent the earliest presentation of primary hyperparathyroidism (PHP). But they may already have bone, renal, and/or cardiovascular disorders. Aim: To evaluate cortical and trabecular differences in hip architecture in normocalcemic (NC) PHP and hypercalcemic (HC) PHP. In both groups we evaluated those 1 year after parathyroidectomy. Materials and Methods: We evaluated a total of 31 patients aged 66.6 years (range, 27.0–80.7 years); 29 women (two postmenopausal [PM]) and two males. The menopause age was 52 years (range, 37–56 years). HC PHP (*n* = 18) and NC PHP (*n* = 13). Hip with the lowest value in *T*‐score was taken to compare by dual‐energy X‐ray absorptiometry (DXA) (Lunar Prodigy Advance): femoral neck (FN), total hip (TH). Cortical and trabecular parameters were assessed by 3D‐Shaper (Galgo Medical). Statistical: *t* test and paired *t* test. Results: Calcemia differs significantly: HC versus NC 10.9 ± 0.56 versus 9.82 ± 0.33; *p* < 0.0001. No differences were observed between groups in age, menopause age, weight, size, DXA, and three‐dimensional (3D) parameters (Table 1). Conclusions: The NC PHP presents similar alterations in the architecture estimated on 3D‐Shaper to HC PHP. Although all 3D parameters evaluated improved after surgery, the trabecular volumetric bone mineral density (vBMD) showed a greater increase. In cortical bone, significant improvement was only observed with 3D Shaper. After surgery patients experienced a significant improvement in TH and some 3D‐Shaper measurements (Table 2).

**Table 1:** 

	HC (*n* = 18)	NC (*n* = 13)	*p*
Trabecular vBMD (mg/cm^3^)	141.9 ± 31.3	138.7 ± 38.2	ns
sDens (mg/cm^2^)	140.6 ± 21.7	138.7 ± 38.2	ns
Cortical thickness (mm)	1.884 ± 0.17	1.850 ± 0.13	ns
TH BMD (mg/cm^2^)	0.870 ± 0.140	0.850 ± 0.140	ns
FN BMD (mg/cm^2^)	0.79 ± 0.11	0.81 ± 0.14	ns

**Table 2:** 

	Before	After	*p*
Trabecular vBMD (mg/cm^3^)	157.19 ± 33.4	160.52 ± 35.0	0.006
sDens (mg/cm^2^)	149.32 ± 22.6	153.2 ± 19.7	0.035
TH BMD (mg/cm^2^)	0.906 ± 0.135	0.922 ± 0.122	0.015
FN BMD (mg/cm^2^)	0.817 ± 0.15	0.846 ± 0.12	0.09

ns = not statistically significant; sDens = cortical BMD.

sDens = cortical BMD.

## Evaluation of denosumab treatment in postmenopausal women's cohort with osteoporosis

### Bayas Robalino SG,^1^ Posadas ML,^2^ Buttazzoni M,^1^ Galich AM ^1^

#### ^*1*^ *Servicio de Endocrinología*

#### ^*2*^ *Departamento de Investigación Clínica, Hospital Italiano de Buenos Aires, Argentina*

Denosumab (Dmab) is an effective treatment for postmenopausal women (PW) osteoporosis. This analysis comprised monocentric data to evaluate the biochemical and densitometric (bone mineral density [BMD]) response and the incidence of adverse effects of Dmab. Methods: Retrospective, cross‐sectional, observational, study in PW >50 years old assigned to Dmab 60 mg biannual for more than 1 year, 2012 to 2017. Statistical analysis: continuous variables with mean and standard deviation were described. STATA software was used for analysis. Results: The population was 514 PW, 71 ± 11 years, body mass index (BMI): 24.3 ± 4.18 kg/m^2^. Previous bisphosphonates (BP) were received in 81%, and 16% were treatment naïve (TN). Mean basal *T*‐score in lumbar spine (LS) was −2.7 ± 0.88 on 96.28% PW and 44% had previous fractures (Fx). 57% had Beta‐CrossLaps (B‐CTx) and it decreased ≥50%. The BMD increased ≥3% in LS in 80% PW, 59% in the total hip (TH) and 56% in the femoral neck (FN). The increase was similar in previous treatments versus TN. Complications: Two (0.38%) PW had jaw osteonecrosis and no atypical femoral fracture was found. Fracture (Fx) was observed in 50 PW (9.72%) during treatment. Vertebral Fx (VFx) in 17 PW, most (*n* = 15) being single vertebra. Post‐suspension Dmab Fx was observed in 41 of 429 PW followed by 2 years after the last dose. VFx was 46.3% and 21.9% hip Fx. The 85% presented on first year after suspension. Single VFx was seen in 79% and multiple VFx in 4 (21%). There were no clinical or biochemical predictive factors. Conclusions: The BMD increase was similar in TN or previous treatments. Post‐suspension Fx incidence was 10%, LS being the most affected. Most occurred in the first year. There was no association with predictor factors, including previous BP. These real‐world data suggest a similar efficacy to the Fracture Reduction Evaluation of Denosumab in Osteoporosis Every 6 Months (FREEDOM) study.

## Tumor‐induced osteomalacia: 10 years of follow‐up

### Vera M,^1^ Buttazzoni M,^1^ Diehl M,^1^ Kitaigrodsky A,^1^ Roitman P,^2^ Ayerza M,^3^ Plantalech L^1^

#### ^*1*^ *Servicio de Endocrinología y Metabolismo*

#### ^*2*^ *Servicio de Anatomía Patológica*

#### ^*3*^ *Servicio de Traumatología, Hospital Italiano de Buenos Aires, Argentina*

Tumor‐induced osteomalacia (TIO) is a rare paraneoplastic syndrome due to increased fibroblast growth factor 23 (FGF23) produced by mesenchymal phosphaturic tumors. When localization is not possible, phosphate and calcitriol are used as treatment. Anti FGF‐23 antibody has been recently approved for TIO. We present a patient with TIO and long‐term follow up. In 2010, A 54‐year‐old woman presented with bone pain and fatigue. X‐rays: bone rarefaction. Laboratory: calcemia 9.4 mg/dL (VR 8.5–10.5), phosphate 1.9 mg/dL (VR 2.5–4.5), parathyroid hormone (PTH) 37.6 pg/mL (VR 8.7–77.1), 25OH‐vitamin D 29 ng/mL (VR >30), alkaline phosphatase (ALP) 1189 IU/L (VR 31–100), RTP 26%. Bone scan: uptake in ribs, metatarsals, and bilateral sacroiliac region. Bone biopsy: hyperosteoidosis. Hypophosphatemic osteomalacia was diagnosed and TIO was suspected. Treatment with phosphates salts (2–3 g/d), calcitriol, and vitamin D3 was started. Bone pain improved, but asthenia persisted. Localization studies were performed without findings (magnetic resonance imaging [MRI], computed tomography [CT], octreotide scan). Genetic studies were negative. In 2018, 68Ga‐DOTATATE positron emission tomography (PET)/CT showed soft tissue uptake in the left paravertebral region. The presence of a tumor attached to the psoas was confirmed by MRI and it was surgically removed. Pathological anatomy: Mesenchymal phosphaturic tumor. Clinical symptoms improved after surgery and serum FG23 was undetectable. She developed hyperparathyroidism associated with chronic phosphate supplementation. Discussion: TIO are often difficult to locate. In this patient, PET/CT with 68Ga‐DOTATATE allowed tumor detection 8 years after the initial diagnosis. Surgical treatment was performed with symptomatic and biochemical improvement. Hyperparathyroidism has been described in patients with long standing phosphate treatment. TIO patients represent a diagnostic and therapeutic challenge.

## Vitamin D levels during fall and winter 2020 in supplemented and non‐supplemented subjects

### Mansur JL,^1^ Ventimiglia F^2^

#### ^*1*^ *Centro de Endocrinología y Osteoporosis La Plata*

#### ^*2*^ *Laboratorio DAgostino‐Bruno, La Plata, Argentina*

The objective was to determine total 25OH‐vitamin D (25OHD) in supplemented and unsupplemented patients during the coronavirus disease 2019 (COVID‐19) pandemic. Patients and methods: Total 25OHD were dosed in 200 subjects. In a subgroup, Ca, P, and parathyroid hormone (PTH) were also determined. A total of 115 patients did not receive supplementation and 85 ingested it. Results: Without supplementation, the subjects were younger (51 versus 63 years) and had a 25OHD level (19.5 versus 35.7 ng/mL, *p* = 0.000). Not supplemented: (1) the 25OHD levels were similar in all ages; (2) 25OHD was lower in subjects with BMI >30 (17.93 ± 7.3 ng/mL); (3) The values in autumn were higher than in winter (24.32 ± 9.3 versus 18.19 ± 6.04 ng/mL). Supplemented subjects: (1) 100.000 IU/month (*n* = 37): 40.86 ± 10.6 ng/mL; (2) 100.000 IU/once in 2 months (*n* = 10): 34.96 ± 15.9 ng/mL; (3) 2.000 IU/day (*n* = 11): 35.17 ± 4.75 ng/mL. In the subgroup with 100,000 IU/month 31 of 37 reached 30 ng/mL. The desirable value (>30 ng/mL) was found in 76% of those supplemented and in 83% of those receiving 100,000 IU/month. The number of subjects deficient, insufficient, and normal were 2, 18, and 65 with supplementation and 62, 44, and 9 without it, respectively. Discussion and Conclusions: The level of 25OHD was studied during 2020, finding differences between those who received supplementation and those who did not. Obesity decreases 25OHD. Older subjects have no lower value. The difference with other works may be due to the fact that in this sample there was no sun exposure.

## Pseudogout after parathyroidectomy in primary hyperparathyroidism

### Komornicki M,^1^ Kitaigrodsky A,^1^ Diehl M,^1^ Buttazzoni M,^1^ Yanzon A,^2^ Plantalech L^1^

#### ^*1*^ *Servicio de Endocrinología, Metabolismo y Medicina Nuclear, Sector Osteopatías Metabólicas, Hospital Italiano de Buenos Aires*

#### ^*2*^ *Cirugía de Cabeza y Cuello, Hospital Italiano de Buenos Aires*

Pseudogout, an acute form of calcium‐pyrophosphate deposition disease, has been observed in patients with primary hyperparathyroidism (PHP). It clinically resembles gout with pain, warmth, swelling, and joint disability, but in this entity the knee is most frequently involved. Pseudogout after parathyroidectomy appears to be triggered by a fall in calcium levels, making the crystals more soluble so they are shed into the synovial space. We report two cases with pseudogout after parathyroidectomy. Case 1: A 57‐year‐old man with medical history of PHP, nephrolithiasis, kidney failure, osteoporosis, and acute pancreatitis a year before. His calcemia was 13.2 mg/dL (NR 8.5–10.5) and parathyroid hormone (PTH) 3541 pg/mL (NR 9–77). X‐rays showed chondrocalcinosis on the knees, pubic symphysis, and wrists. A 4 × 1.5‐cm^2^ parathyroid adenoma was removed. The calcemia fell to 8.7 mg/dL at the third day. He developed acute arthritis first in the right and then in the left knee, that solved with corticosteroids. Case 2: A 41‐year‐old man was admitted for severe hypercalcemia. Backgrounds: Kidney failure and left ankle fracture. Calcemia was >14 mg/dL and PTH 1026 pg/mL. He was treated with hydration and bisphosphonates; calcium decreased up to 10.2 mg/dL. He suffered acute arthritis on the right ankle. A 6 × 2.5‐cm^2^ parathyroid adenoma was resected later, and he evolved with normocalcemia. Two weeks later arthritis appeared in the right knee. The arthrocentesis diagnosed pseudogout. He received local corticosteroids and relief symptoms. Discussion: Pseudogout should be suspected in patients with acute arthritis after parathyroidectomy. In these patients, pseudogout manifested beyond the treatment of hungry bone syndrome and normocalcemia. This complication was more frequently in the past, possibly due to the earlier diagnosis of PHP nowadays.

## Management of hip fracture during the COVID‐19 pandemic

### Buttazzoni M,^1^ Boietti B,^2^ Kitaigrodsky A,^1^ Komornicki M,^1^ Vera ME,^1^ Slullitel PAI,^3^ Carabelli G,^3^ Barla J,^3^ García Barreiro G,^3^ Benchimol J,^2^ Diehl M^1^

#### ^*1*^ *Servicio de Endocrinología, Metabolismo y Medicina Nuclear, Sección de Osteopatías Metabólicas*

#### ^*2*^ *Servicio de Clínica Médica*

#### ^*3*^ *Servicio de Traumatología y Ortopedia, Hospital Italiano de Buenos Aires, Argentina*

Hip fracture (HF) is associated with a higher risk of new fractures and mortality. The impact of the coronavirus disease 2019 (COVID‐19) pandemic on HF patients in Argentina is unknown. This study aimed to compare treatment and outcomes after HF during the pandemic versus the same period in 2019. Methods: Observational retrospective study comparing 2020 (03/20/2020–07/20/2020) and 2019 (03/20/2019–03/20/2019) cohort. Data were obtained from the institutional HF registry and the electronic medical record. Clinical frail scale and Charlson index were assessed. In 2020 in‐hospital bisphosphonate (BP) use and telemedicine were implemented. Bivariate analysis of qualitative variables was performed with χ^2^ or Fisher, and for quantitative variables, *t* test or Wilcoxon. STATA 14 software was used. Results: Both cohorts (2019 *n* = 113, 2020 *n* = 74) were similar in age (88 versus 86.5 years), sex, body mass index (BMI), previous fractures, osteoporosis treatment, fracture type, parathyroid hormone (PTH), and vitamin D, but the 2020 cohort presented greater frailty (*p* < 0.0001) and Charlson index (*p* < 0.0001). COVID test was negative in all patients. Median time to surgery was 1 (1–2) days (*p* = 0.9). In‐hospital endocrinological evaluation was performed in >80% (*p* = 0.61). BP treatment was 30% in 2019 versus 54% in 2020 (*p* = 0.009). Zoledronic acid (ZOL) was used in 85% of cases without severe adverse effects. Endocrinological follow‐up was 25% in the 2019 cohort versus 43.1% (telemedicine) in 2020 (*p* = 0.01). No significant differences in hospital stay, new fractures, readmissions, and 3‐month mortality were found. Discussion: Surgical and in‐hospital evaluation were similar during COVID pandemic. Postoperative ZOL was safe and increased access to treatment. Telemedicine improved follow‐up. Length of hospitalization, readmissions, and 3‐month mortality were similar.

## Primary hyperparathyroidism in pregnancy during the COVID‐19 pandemic

### Komornicki M, Buttazzoni M, Diehl M, Mazzaro E, Alonso G, Varela F, Vallone M, Kitaigrodsky A

#### Hospital Italiano de Buenos Aires, Argentina

Primary hyperparathyroidism (PHP) in pregnancy is infrequent. A retrospective series found that it occurred in 0.03% of women in reproductive age who underwent routine screening of serum calcium. There are risks to the fetus and neonate including miscarriage and PHP. We report a case of PHP diagnosed in postpartum and the newborn follow‐up. Case: A 40‐year‐old woman 37 weeks pregnant was admitted in April 2020 due to labor. She had medical history of systemic lupus erythematosus and kidney failure diagnosed in the third trimester, treated with hydroxychloroquine and corticosteroids. Calcemia 12.3 mg/dL (NR 8.5–10.5), phosphate 3.8 mg/dL (NR 2.5–4.5), creatinine 3.52 mg/dL, 25(OH)D 19 ng/mL, and parathyroid hormone (PTH) 350 pg/mL (NR 9–77). Ultrasound and parathyroid scintigraphy revealed a coincident image. A 2.3 × 1.5‐cm^2^ parathyroid adenoma was removed. The newborn (male) calcemia was 7.1 mg/dL, PTH 7 pg/mL, and 25(OH)D 15 ng/mL. He was asymptomatic. He received treatment with intravenous calcium, and then with oral calcium, calcitriol, and vitamin D. Over the days, the requirement decreased. Hypoparathyroidism finally reversed at 3 weeks. Discussion: In this puerperal woman surgical resolution was decided considering that hypercalcemia could aggravate kidney failure. Decreased kidney function and breastfeeding were other difficulties for medical treatment. The bone impact of PHP was also considered, together with corticosteroids use and high bone remodeling of pregnancy and lactation. Neonatal hypoparathyroidism is a rare complication. There are few case reports, mostly transitory as in our case, with scarce long‐term follow‐up data. The treatment of PHP in pregnancy and puerperium constitutes a challenge. Other difficulties were added in this case due to quarantine and the coronavirus disease 2019 (COVID‐19) pandemic.

